# Emotion Analysis of Telephone Complaints from Customer Based on Affective Computing

**DOI:** 10.1155/2015/506905

**Published:** 2015-11-08

**Authors:** Shuangping Gong, Yonghui Dai, Jun Ji, Jinzhao Wang, Hai Sun

**Affiliations:** ^1^Language and Culture Research Institute, National University of Defense Technology, Changsha 410074, China; ^2^School of Information Management and Engineering, Shanghai University of Finance and Economics, Shanghai 200433, China; ^3^Department of Operation Quality and Service Administration, China Unicom Co. Ltd., Shanghai Branch, Shanghai 200070, China; ^4^School of Management, Fudan University, 220 Handan Road, Shanghai 200433, China

## Abstract

Customer complaint has been the important feedback for modern enterprises to improve their product and service quality as well as the customer's loyalty. As one of the commonly used manners in customer complaint, telephone communication carries rich emotional information of speeches, which provides valuable resources for perceiving the customer's satisfaction and studying the complaint handling skills. This paper studies the characteristics of telephone complaint speeches and proposes an analysis method based on affective computing technology, which can recognize the dynamic changes of customer emotions from the conversations between the service staff and the customer. The recognition process includes speaker recognition, emotional feature parameter extraction, and dynamic emotion recognition. Experimental results show that this method is effective and can reach high recognition rates of happy and angry states. It has been successfully applied to the operation quality and service administration in telecom and Internet service company.

## 1. Introduction

Customer service has been playing an increasing important role in the competitive market in business administration in recent years. As one of the routines in customer service, the handling of telephone complaints from customers plays a significant role in showing the image of the enterprise, obtaining the feedback from the market, and improving the loyalty of the customers. Therefore, it has attracted high attention from enterprises and researchers. The existing researches on customer complaints mainly focus on the classification of complaints, record analysis, handling operation, information management, and so on [1–3]. However, less attention has been paid to the technical and intelligent analysis of the complaint speeches so far. Actually, the customer's speeches in telephone complaints usually carry rich emotions and provide valuable information for perceiving the customer's degree of satisfaction and studying the complaint handling skills.

Affective computing was proposed by Professor Picard in 1977, attempting to create a way of perception, recognition, and understanding of human emotion, which would make the computer system intelligent and sensitive so as to react friendly to human emotions [4]. It has become a burgeoning area of research in human-computer interaction during the past decades. Up to now, scholars have presented a lot of methods and models to deal with the affective computing issues from speech signals [5]. However, most researches are confined to isolated speeches and contain only one type of emotion [6–8]. The speeches in the telephone complaint of a real case are more complicated because they are embedded in the conversations between the service staff and the customer and are embodied with the dynamic changes of emotions ranging from excitement to calmness [5, 9]. Therefore, an effective technology, which is independent of the speakers' emotional changes [10], should be first utilized to precisely distinguish the customer's speeches from those of the service staff's. On this basis, the conversations in telephone complaints can be segmented into separate speeches according to their different speakers. In the recognition of customer's emotions, the computing method ought to consider the dynamic changes of emotions in customer's continuous speech as well as the possible noise in telephone communication [5].

In this paper, we first study the characteristics of telephone complaint speeches and then conduct a cost-sensitive learning technology [10] to identify the different speakers and separate their speeches from the conversations. Thereafter, a robust method and signal process are proposed to recognize the customer's changing emotions. Furthermore, affective computing technology is explored by using support vector machine (SVM) to process the extracted MFCC parameters. The proposed method and technology have been successfully applied to the administration of telecom and Internet services.

This paper is organized as follows. In Section 2, theory and methodology are introduced as the basis of our research work; in Section 3, the characteristics of telephone complaint speeches are displayed and the speaker identification from conversations is discussed; in Section 4, the analysis method based on affective computing technology is proposed to recognize the dynamic changes of customer's emotions in telephone complaints; in Section 5, an experiment is illustrated to show the performance of the presented method; Section 6 is the conclusion and discussion of this paper.

## 2. Theory and Methodology

### 2.1. Emotion Classification and Description

The classifications and the descriptions of emotions have been so diverse due to the different understanding of people's psychological experiences in a variety of applications. For a long time, scholars have not reached consensus on the classification of emotion. However, two types of emotion classifications have been widely accepted in psychological studies: one is the classification of basic emotions, and the other is the description of emotion in dimensions [11].

The basic emotion theory claimed that each type of emotions has its basic and disparate characteristics in human experiences, physiological arousal patterns, and the explicit modes. It suggested that all human complex emotions are the different combinations of basic emotions. And Ortony and Turner (1990) summarized the typical classifications of basic emotions proposed by the scholars in this field, which are shown in Table 1 [12].

**Figure 1:**
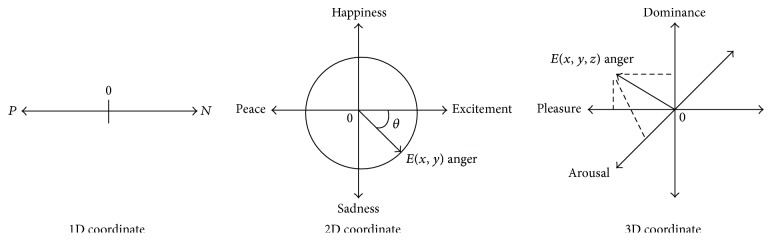
Continuous form of emotions in different dimensions.

Different from the basic emotion theory, dimension theory argued that the changes of human's emotions are continuous emotion. It suggests that emotions should be described in a dimensional space, and the similarities and differences between each emotion depend on the dimensions in the space distance. Russell and Peterson proposed the two-dimensional circumplex model for the sentiment classification, which included pleasant-unpleasant dimension and the strength dimension. They thought that affective states can be described by the above two dimensions [13–15]. In addition, Wundt put forth the three-dimensional theory of emotion [16], and he proposed that each emotion is one part of a continuum and different emotions are mapped to specific points in a space with three dimensions, among which, P (Pleasure-Displeasure) dimension reflects a positive or a negative evaluation such as comfortable or not comfortable and agreeable or disagreeable. A (Arousal-Nonarousal) dimension reflects the degree of physiological stimulation and takes some action preparations, which might be active or passive. D (Dominance-Submissiveness) dimension can reflect the strength and the desire for the control of a speaker; it accounts for dominant or submissive position. The continuous form of emotions in different dimensions is shown in Figure 1 [9].

### 2.2. Acoustic Parameters Related to Emotions

The commonly used acoustic parameters related to emotions can be divided into three categories [17], prosody parameters, spectral parameters, and sound quality parameters. In the above categories, prosody parameters such as the duration, pitch, and energy of a speech signal are the basic parameters for emotion recognition. Mel Frequency Cepstrum Coefficient (MFCC) parameters usually perform better than the other spectral parameters and are widely applied to speech recognition [18]. Sound quality parameters such as format frequency and bandwidth are effective in differentiating the emotions associated with attitudes and intentions [19].

Recent experiments have shown that the combined parameters from different categories can acquire a more ideal performance [9, 19]. For example, the combination of short-time energy, pitch, short-time zero crossing rate, first formant, second formant, voice speed, number of voice breaks, and 12-order MFCC parameters was successfully applied to the dynamically affective computing on vocal social media [9]. In order to get the dynamic recognition of customer's emotions, the choice of acoustic parameters should take into account both affective features and voice features. Based on the previous research results, we adopt the short-time average energy, short-term zero crossing rate, pitch, formant, and 12-order MFCC coefficients as the feature parameters for speaker identification and emotion recognition in the conversations of telephone complaints.

Short-time average energy refers to the average energy of the speech signal. It is mainly used for acoustic boundary and the ligatures boundary. Short-time average energy can be expressed as in the following formula:

(1) $$\begin{array}{c} E_{n}=\overset{N-1}{\underset{i=0}{\sum }}x_{n}^{2}\left(\;i\;\right). \end{array}$$

Short-time zero crossing rate refers to signal through the zero frequency in a frame. And it can be expressed as in the following formula:

(2) $$\begin{array}{c} Z_{n}=\overset{N-1}{\underset{i=0}{\sum }}\mathrm{sgn}\left[\;x_{n}\left(\;i\;\right)\;\right]-\mathrm{sgn}\left[\;x_{n}\left(\;i-1\;\right)\;\right]. \end{array}$$

**Figure 2:**
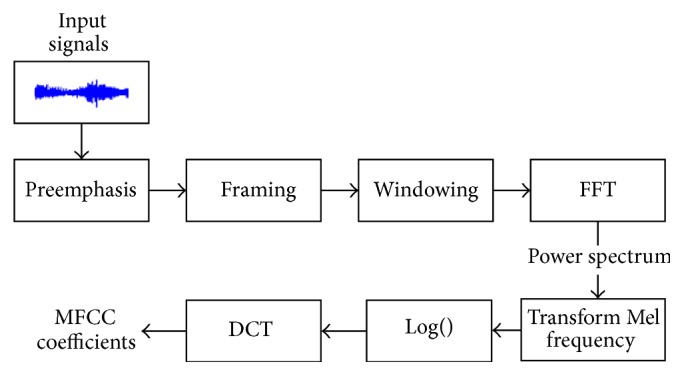
Extraction process of MFCC coefficients.

MFCC is derived from a type of cepstral representation of the audio clip. It reflects the characteristics of the short-time amplitude spectrum of speech. Extraction process of MFCC coefficients is shown in Figure 2.

### 2.3. Speech Emotion Recognition Algorithm

The main method of speech recognition includes *k*-nearest neighbor method (*k*-NN), artificial neural network (ANN), hidden Markov model (HMM), Gaussian mixture model (GMM), and support vector machine (SVM). Telephone complaints are continuous speeches in the conversations between the customer and the service staff with background noise. Among the proposed methods, ANN can simulate the complicated relationship between the input and output variables and utilize the hidden knowledge very well by sufficient training samples, while SVM has the advantages of superior stability, good generalization ability, and high efficiency [10, 20]. We will compare the performances of ANN with those of the SVM methods in our study.

#### 2.3.1. Artificial Neural Network

A neural network is composed of a number of nodes, or units, connected by links. Each link has a numeric weight associated with it. Weights are the primary means of long-term storage in neural networks, and learning usually takes place by updating the weights. Some of the units are connected to the external environment and can be designated as input or output units.

**Figure 3:**
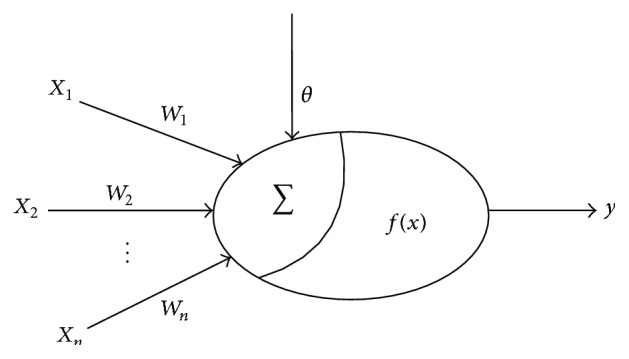
The simplified structure of neurons.

Back-Propagation Neural Network (BPNN) is one of the most widely used artificial neural networks, and it adopts a kind of error back-propagation algorithm for training multilayer feed forward neural network. Therefore, it can learn and store the input-output mapping relationships. BPNN is based on gradient descent method which minimizes the total of the squared errors between the actual and the desired output values. In BPNN, the basic units of neural network are artificial neuron, which are simulating biological neurons with the simplified structure as shown in Figure 3.

The simplified structure neuron is a nonlinear element of a multiple input and a single output, and its relationship can be described as

(3) I=∑i=1nwixi−θ,y=fI,

where *x*_*i*_ is the input value, *θ* is a threshold, and *w*_*i*_ means the strength of weight. *f*(*x*) is the excitation function. Typically, a BPNN topology structure includes input layer, hidden layer, and output layer.

#### 2.3.2. Support Vector Machine

Support vector machine is based on the statistical learning theory and VC dimension theory which is the structural risk minimization principle. Through a nonlinear mapping, SVM method takes the sample space to map a high dimensional feature space and makes nonlinear separable problem in the original sample space changed as a linear separable problem in the feature space.

When we use the SVM method for emotion recognition, the selection of kernel function is very important. Four kinds of kernel function are often used in SVM, which can be described as follows.

(i) Linear kernel function: *K*(*x*, *y*) = *x* ^*T*^ · *y*.
(ii) Polynomial kernel function: *K*(*x*, *y*) = [(*x* · *y*) + 1]*d*.
(iii) RBF kernel function: *K*(*x*, *y*) = exp⁡(−‖*x* − *y*‖^2^/2*σ* ^2^).
(iv) Sigmoid kernel function: *K*(*x*, *y*) = tanh⁡(*a*(*x* · *y*) + *b*).

## 3. Telephone Complaints and Speaker Identification

### 3.1. Characteristics of Telephone Complaints

The speeches of telephone complaints occur in the conversations between the service staff and the customer. Therefore, the speakers should be identified so that we could find out the customer's speeches and deal with the emotion recognition of his speeches. In order to do well in spell recognition, the speeches in conversations will be first cut into a series of segmentations according to their continuous sound waves. Usually, the speech signals contain possible noises in telephone communication and the speaker's surroundings, so a bandwidth limited filter and the 50 Hz circuit noise elimination had to be adopted in the preprocessing procedure [5]. Through the careful analysis of a large number of complaints, we found that calmness, discontent, and anger are three typical emotional states which can satisfy the requirements of service management. Therefore, we will mainly discuss the recognition of these three typical emotions.

Our previous study has indicated that intensity, pitch frequency, and spectrum parameter could be used as the prominent feature parameters for distinguishing those emotional states [5]. For example, someone's speech characteristics in three emotional states (calmness, discontent, and anger) are shown in Table 2.

From Table 2, we can find that calmness has the lowest mean-intensity with pitch range below 170 Hz. Discontent and anger have stronger mean-intensities, and their pitch ranges expand to more than 280 Hz. In particular, the pitch range of anger reaches more than 300 Hz.

### 3.2. Speaker Identification from Conversations

**Figure 4:**
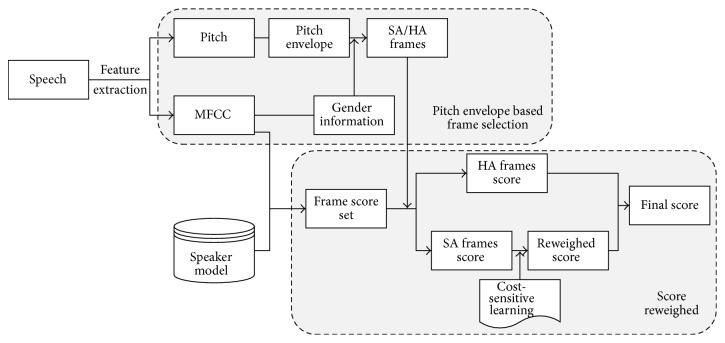
Speaker identification algorithm based on cost-sensitive learning technology.

In order to precisely distinguish the customers' speech from the conversations, we adopted a robust speaker identification algorithm. The algorithm introduced a cost-sensitive learning technology to reweight the probability of the tested affective utterances in the pitch envelope level, which can effectively enhance the robustness in emotion-dependent speaker recognition as shown in Figure 4 [10].

## 4. Framework of Recognition

**Figure 5:**
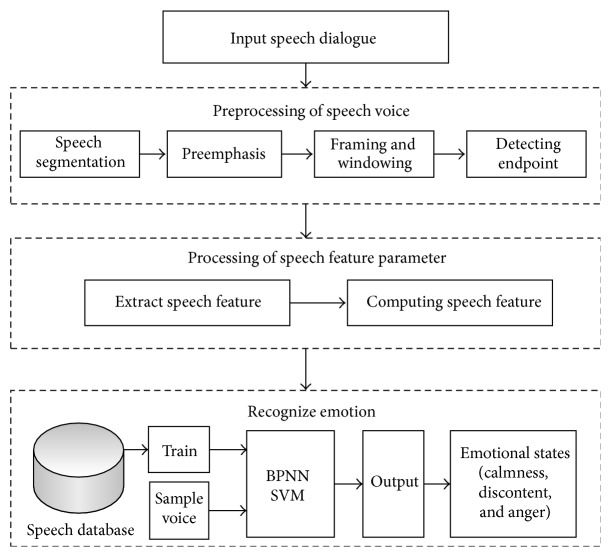
The process of speech emotion recognition.

Based on previous analysis, we propose a recognition method of customer's emotions in telephone complaints as shown in Figure 5.

The recognition process includes preprocessing, feature parameter extraction, and emotion recognition.

### 4.1. Preprocessing of Speech Signals

In order to improve the quality of speech signals, the preprocessing aims to provide successor analysis services for feature extraction and speech emotion recognition, which may include speech unit segmentation, preemphasis, framing and windowing, and detecting endpoint [21–23].

#### 4.1.1. Characteristics of Telephone Complaints

Because the high frequency part of the spectrum is relatively small in telephone voice signals, the preemphasis processing is usually utilized to enhance the high frequency part of the signals' amplitudes. This is frequently dealt with by the first order high pass filter as shown in the following:

(4) $$\begin{array}{c} H\left(\;z\;\right)=1-az^{-1}. \end{array}$$

#### 4.1.2. Framing and Windowing

Previous studies have found that the characteristics and physical characteristic parameters of telephone speeches can remain stable in the period of 10 ms–30 ms and can keep the short-time stationary [22, 24]. Therefore, it is necessary to split the speech signals into the time periods so as to analyze them based on the smallest units. This is processed by a frame with the length of 10 ms–30 ms.

Windowing is to select the window function after framing, and there are two factors to be considered, namely, the shape and the length. Generally speaking, we use three windows: rectangle window, Hamming window, and Hanning window.

The rectangular window is shown as in the following formula:

(5) $$\begin{array}{c} w\left(\;n\;\right)=\left\{\begin{array}{cc} 1, & 0\leq n\leq N-1\\ 0, & \text{others.} \end{array}\right. \end{array}$$

Hamming window (Hamming) is shown as in the following formula:

(6) $$\begin{array}{c} w\left(\;n\;\right)=\left\{\begin{array}{cc} 0.54-0.46\cos\left[\frac{2\pi n}{N-1}\right], & 0\leq n\leq N-1\\ 0, & \text{others.} \end{array}\right. \end{array}$$

Hanning is shown as in the following formula:

(7) $$\begin{array}{c} w\left(\;n\;\right)=\left\{\begin{array}{cc} 0.5\left[1-\cos\left(\frac{2\pi n}{N-1}\right)\right], & 0\leq n\leq N-1\\ 0, & \text{others.} \end{array}\right. \end{array}$$

#### 4.1.3. Endpoint Detecting

The main purpose of endpoint detecting is to use computer technology and digital processing to find the start point and the end point of emotional information contained in a section of speech signal. The basic parameters of the endpoint detecting are short-time energy, short-time average zero crossing rate, and short-time correlation function, and so forth. After detecting endpoints, we will employ the speaker identification algorithm to identify the customer's speeches and put them into the next step for feature parameter extraction.

### 4.2. Emotional Feature Parameter Extraction

Research findings in the fields of psychology and metrics have pointed out that prosody and voice quality in speeches are the most intuitive indicators to reflect the changes of a speaker's emotions. Statistical analysis shows that if someone is happy, he usually speaks very fast and the volume is high. However, if he is in time of sadness, he tends to speak slowly with relative small intensity. The emotional features which are commonly used in the researches of speech signals include short-time average energy, short-term zero crossing rate, pitch frequency, formant parameters, and Mel Frequency Cepstrum Coefficient (MFCC), as well as a variety of their derived variant forms, such as maximum, minimum, mean, range, and change of the covariance rate [25, 26]. In our study, we adopt MFCC parameters as the emotional features because they have very good ear perception features and contain the comprehensive characteristics of speech emotions. Besides, Mel scale has the advantages of simple calculation and easy distinguishing.

The calculation of Mel frequency is shown in the following formula:

(8) $$\begin{array}{c} \text{Mel}\left(\;f\;\right)=2595\;\text{lg}\left(\;1+\frac{f}{700}\;\right). \end{array}$$

### 4.3. Emotion Recognition

As we discussed above, the telephone complaints of a real case are included in the conversations between the service staff and the customer, so we should firstly identify the speakers. And it is performed by the algorithm based on cost-sensitive learning technology.

After detecting the customer's speeches, we adopted the affective computing technology based on BPNN and SVM methods and recognized the dynamic changes of customer's emotions from the extracted feature parameters of his complaints.

In the management of customers' telephone complaints, the typical emotions frequently concerned by the service staff are anger, discontent, and calmness. Anger and calmness are not likely to occur simultaneously; then, discontent can be regarded as their intermediate state. Therefore, anger, discontent, and calmness can reflect the customer's possible emotion changing period in a conversation and can be used to evaluate the service effects in telephone complaint management. In speech recognition algorithm, we used BPNN, SVM methods and cost-sensitive learning technology [10] to optimize recognition rate.

## 5. Experiment and Results

### 5.1. Experiment Data

We took CASIA Chinese speech emotional database and the records from the customer complaint service center of a telecom and Internet service company as experiment data. CASIA was developed by the National Laboratory of Pattern Recognition and Human-Computer Interaction research group at Chinese Academy of Sciences Institute of Automation [8]. It has been widely used as the standard corpus for Chinese language test. In this corpus, each speech with the same semantic texts is spoken by 2 men and 2 women in six different emotional tones: happy, sad, angry, surprise, fear, and neutral. Therefore, it can be used to evaluate the reliability and validity which are only related to the emotions [9].

The records from the customer complaint service center are 252 real speech samples saved as wav files at the sampling rate of 16 kHz. Each sample contains a whole conversation process between the staff and the customer with 6–12 sentences, which includes the dynamic changes of different emotional states.  Table 3 shows the part of “.wav” files that we will mention in the following discussion.

**Figure 6:**
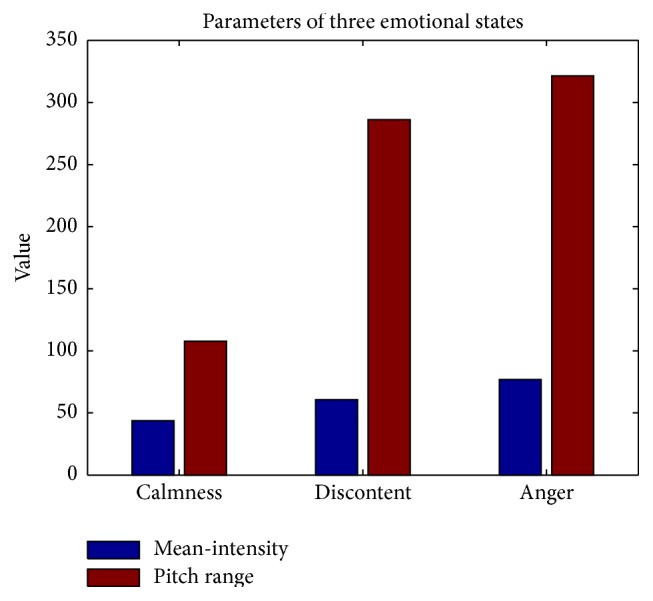
The characteristic value of three emotional states.

Figure 6 shows the characteristic value (mean-intensity and pitch range) of some “.wav” file from Table 3. It can be seen that the values of anger state are much higher than those of calmness state.

**Figure 7:**
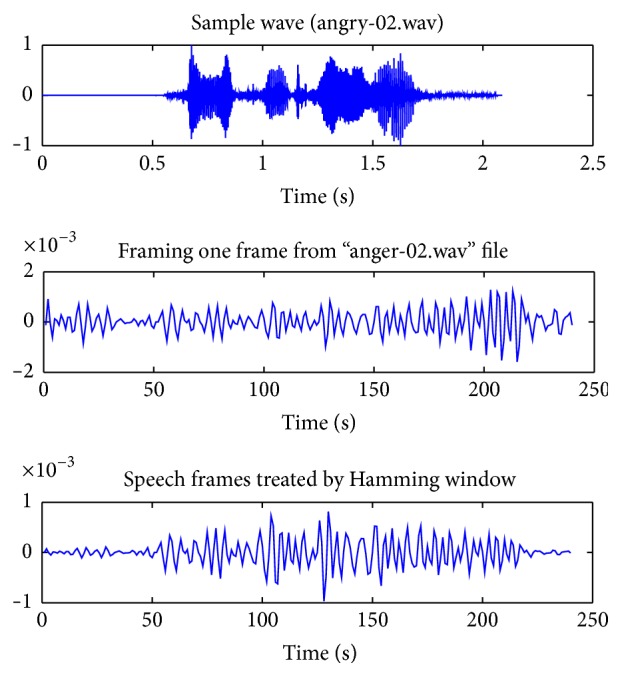
Framing and Hamming windowing of speech file.

In order to suppress the noises and highlight the main features of the speeches, framing and windowing are used to preprocess the samples and transfer the speech signals into frames. For example, the framing and Hamming windowing waveform of file “Anger-02.wav” whose frame length is 240 and the shift is 80 is as shown in Figure 7.

### 5.2. Feature Extraction

**Figure 8:**
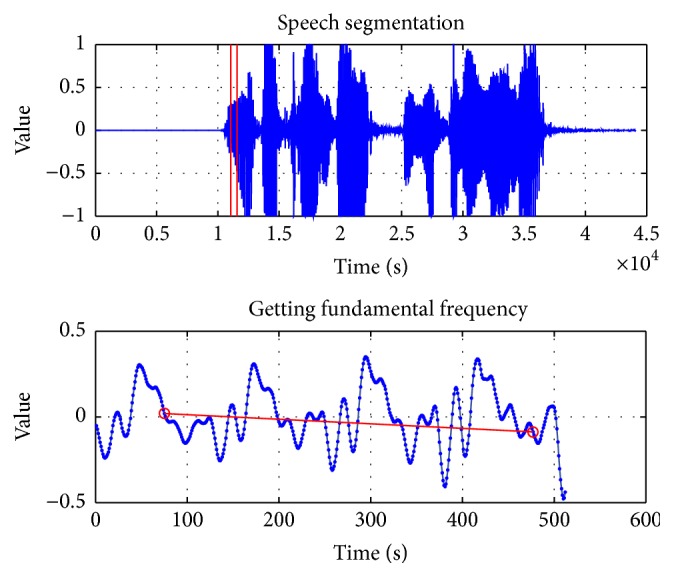
Fundamental frequency of the sample voice.

The intensity represents the strength of voice by the amplitude of speech signals. We considered the typical complaint situations such as the complaints of broadband fault and fee deduction of rubbish short messages. For example, “My home broadband has gone wrong. When would you repair it?” Getting fundamental frequency from this voice file is as shown in Figure 8.

**Figure 9:**
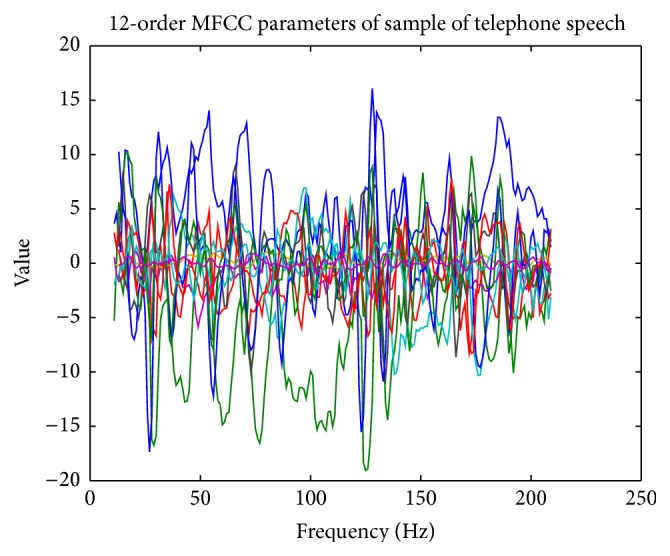
12-order MFCC parameters extracted from a sample of telephone speech.

The 12-order MFCC parameters extracted from the above sample speech are shown in Table 4 and Figure 9.

### 5.3. Recognition Results

After passing the test by CASIA database, we apply our method to the 252 real speech samples [5]. Each sample includes a dynamic conversation between the service staff and the customer. The three recognition methods results were shown in Table 5.

From Table 5, we can find that SVM with combined 12-order MFCC and short energy has the highest average recognition rate. It can reach 88.60%, 61.83%, and 89.80% in the dynamic recognition of calmness, discontent, and anger, respectively. The results also show that calmness is not a prominent emotional state and may be the neutral description of customer's psychological states. The proposed method has been successfully applied to the operation quality and service administration in telecom and Internet service company.

## 6. Conclusion and Discussion

This paper studied the characteristics of customers' telephone complaint speeches and discussed the factors of speaker identification from the conversations as well as the dynamic emotion recognition of customer speeches. In order to recognize the dynamic emotions in customers' complaints, we first employed the cost-sensitive learning technology to identify the speakers and then utilized the BPNN and SVM methods to realize the recognition of customers' dynamic emotions based on the affective computing from extracted feature parameters. Experimental results show that SVM with combined 12-order MFCC and short energy has the highest average recognition rate of 80.08%.

In the recognition of speech emotions, six typical emotions, namely, anger, distaste, fear, joy, sadness, and surprise, have been well researched based on the isolated speeches. Nwe et al. obtained the accuracy rate of 78% by HMM method [27]. Bhatti et al. developed a modular ANN and reported the correct rate of 83% [28]. The hybrid SVM method with combining acoustic features and linguistic information presented by Schuller et al. can achieve higher accuracy rates than HNN and ANN methods [29]. Emotion recognition from continuous conversations has been a new research issue in recent years [9, 30]. However, there are few studies on telephone complaints. Generally speaking, the reliable recognition rate may be 70%–80% [9].

Due to its specificity and complexity, the dynamic emotion analysis of customers' telephone complaints in the real application is expected for further researches. This paper provides a valuable reference on this issue. In the further improvement, the feature parameters which reflect the calm state may be explored and the quantitative evaluation should be made to show the strength and its dynamic changes of emotions. Besides, a knowledge base considering the differences of the customer's gender, age, and other attributes may be introduced to enhance the recognition rate.

## Figures and Tables

**Table 1:** Classifications of basic emotions.

Scholars	Classifications of basic emotions
Arnold	Anger, aversion, courage, dejection, desire, despair, fear, hate, hope, love, and sadness

Ekman, Friesen, and Ellsworth	Anger, disgust, fear, joy, sadness, and surprise

Frijda	Desire, happiness, interest, surprise, wonder, and sorrow

Gray	Rage and terror, anxiety, and joy

Izard	Anger, contempt, disgust, distress, fear, guilt, interest, joy, shame, and surprise

James	Fear, grief, love, and rage

McDougall	Anger, disgust, elation, fear, subjection, tender-emotion, and wonder

Mowrer	Pain, pleasure

Oatley and Johnson-laird	Anger, disgust, anxiety, happiness, and sadness

Panksepp	Expectancy, fear, rage, and panic

Plutchik	Acceptance, anger, anticipation, disgust, joy, fear, sadness, and surprise

Tomkins	Anger, interest, contempt, disgust, distress, fear, joy, shame, and surprise

Watson	Fear, love, and rage

Weiner and Graham	Happiness, sadness

**Table 2:** Someone's speech characteristics.

Speech characteristics	Someone's emotional states		
	Calmness	Discontent	Anger
Mean-intensity (*μ*v)	43.82	60.59	76.82
Maximum pitch (Hz)	315.59	408.13	532.11
Min pitch (Hz)	148.61	122.05	180.69
Mean pitch (Hz)	207.82	257.33	267.91
Pitch range (Hz)	107.77	286.08	321.42

**Table 3:** Part of the “.wav” files of telephone complaints.

Recorders of telephone complaints		
(1) Anger 01-broadband fault.wav	(4) Discontent 01-broadband fault.wav	(7) Calmness 01-broadband fault.wav

(2) Anger 02-improper charges.wav	(5) Discontent 02-improper charges.wav	(8) Calmness 02-improper charges.wav

(3) Anger 03-harassing messages.wav	(6) Discontent 03-harassing messages.wav	(9) Calmness 03-harassing messages.wav

**Table 4:** 12-order MFCC parameters.

12-order MFCC coefficients						
1	11.9380	13.8424	14.5935	12.0397	⋯	16.7550
2	−3.6532	−1.2277	−0.5424	−0.9713		5.7762
3	−1.2243	−0.3909	1.0603	0.4253		3.1929
4	0.0357	1.8983	0.4281	−0.6828		7.2475
5	0.3830	0.7901	0.2184	−0.4252		0.3533
6	−0.6169	−0.2753	−0.0506	−0.3335		−0.3407
7	1.3436	0.6748	−1.2993	−2.5699		1.1169
8	1.6690	3.4504	4.5318	4.7105		1.6278
9	−1.6734	1.3769	3.5090	4.7951		−1.5150
10	−0.3022	−0.6043	−0.8377	−0.3965		−0.4561
11	−0.9944	−1.0738	0.5673	1.1786		2.0980
12	0.2397	0.3336	0.0626	−0.1852		0.4053

**Table 5:** Average recognition rate of emotions.

Recognition methods	Recognition rate			
	Calmness	Discontent	Angry	Average
BPNN	81.22%	60.46%	80.92%	74.20%
SVM (12-order MFCC)	84.50%	61.40%	83.27%	76.39%
SVM (combined 12-order MFCC and short energy)	88.60%	61.83%	89.80%	80.08%
